# Rural Health at a Crossroads: How Policymakers Have Failed Rural America and What Can Be Done for a Healthier Tomorrow

**DOI:** 10.1111/1468-0009.70106

**Published:** 2026-06-10

**Authors:** MICHAEL E. SHEPHERD

**Affiliations:** ^1^ School of Public Health University of Michigan

**Keywords:** rural health, Medicaid, Medicare, telehealth, mobile health

## Abstract

**Policy Points**

- Rural communities have more limited access to medical care and worse health outcomes.
- Existing federal and state policies largely fall short in addressing rural access disparities.
- Rural health woes require increased government funding for providers and the embrace of alternative health delivery mechanisms, as well as expanded public‐private/nonprofit partnerships to overcome access barriers.

**Context:**

Rural and urban areas have diverged significantly in health care access and health outcomes over the last four decades. Federal and state policies have played important roles in shaping these trends.

**Methods:**

I qualitatively review previous and existing federal and state policies that have shaped contemporary rural‐urban health disparities. I review academic literature on recent policy enactments that have shown promise for alleviating some rural health struggles as well as scholarship on how the politics of rural health have stymied better policymaking.

**Findings:**

Large scale federal investments (e.g., the Hill‐Burton Act) were required to bring modern health care to rural communities in the first place, but inadequate and nonuniversal policies enacted since the 1970s and the increasing corporatization of health care over the last decades have led to withering rural health care access. The recent “One Big Beautiful Bill Act” will likely make these matters worse. Policies that have expanded telehealth and mobile health access, as well as subsidized rural transportation services and changes to public payer reimbursement policies, have provided some optimism. However, the broader politics of rural health limit policy opportunities.

**Conclusion:**

Rural health has suffered, in part, due to state and federal policy failures. While some incremental changes have certainly shown evidence of potential improvements, a more radical policy agenda may be needed to maintain or improve health care access in rural communities. There is mixed‐to‐negative evidence regarding whether the political environment will allow for sufficient policy improvements.

Rural and urban americans increasingly experience different realities socially and politically.^1^, ^2^ The divergence in health care access in rural parts of the country from urban and suburban areas has been just as seismic.^3^, ^4^ This access gap contributes to disparate chronic disease struggles, drug addiction, mental health challenges, and worse overall health. As a result, rural Americans live roughly three years shorter lives on average than urban Americans, with some experiencing declining life expectancy since the turn of the century.^5^, ^6^, ^7^, ^8^ While many of the health difficulties facing rural Americans have been attributed to factors like “rural culture,” or simply demographics, inadequate health policy related to access has played a critical role in shaping rural disparities.^9^, ^10^

Today, rural health is at a crossroads. The challenges facing rural communities are mounting at the exact time in which policymakers’ concerns with the plight of rural communities are seemingly at an all‐time high and major, albeit mostly negative, policy changes are being proposed and enacted. Despite many negative trends in rural health, some policymakers and health care organizations have made considerable advancements in turning the tide on rural health in recent years. Investments in telehealth, mobile health units, and partnerships between nonprofit hospitals and community organizations provide room for optimism. However, a cohesive and comprehensive rural health policy agenda and substantial government investments are still necessary to address the many health needs of rural American communities. Importantly, the politics surrounding rural communities poses significant challenges to overcoming contemporary rural health challenges.

## Policies That Have Failed to Deliver

Federal investments in rural health infrastructure during and following the New Deal, such as those stemming from the Hill‐Burton Act (1946‐1974), brought modern medical care within reach of many rural Americans for the first time.^11^, ^12^ As much as these large federal actions were responsible for expanding health care access to rural communities, it has been the retrenchment of these investments and other insufficient federal and state policies that have contributed to modern rural health access struggles in rural America in recent years.

The access disparities that plague rural America today are driven by the interaction between inadequate and poorly designed policies and the realities of rural demographics. Rural communities, by definition, have smaller populations and, thus, lower and more sporadic patient volumes. Due to the relatively lower incomes and older ages of rural people, rural Americans are disproportionately likely to be covered by Medicaid and Medicare.^13^, ^14^ As a result of the fee‐for‐service models and lower reimbursement rates of these programs relative to private insurance, as well as relatively higher operating costs, rural health care providers are often financially instable.^15^, ^16^ Additionally, and especially in states that failed to expand Medicaid under the Affordable Care Act (ACA), larger shares of rural populations lack health insurance. This lack of insurance contributes to larger “uncompensated care” loads for rural providers, further imperiling the finances of providers and often leading them to abandon rural communities.^17^, ^18^ Demographics that lead to disproportionate reliance on public programs that underpay, along with higher fixed operating costs for providers, have created seemingly perpetual financial strain. Policymakers have attempted to remedy these issues with a variety of tax subsidies and special rural hospital designations. However, the incomplete nature of these policies has not alleviated substantial financial difficulties.^16^

Medicaid expansion under the ACA drastically reduced the number of rural people without insurance and improved the finances of rural providers.^17^ However, many states with large rural populations, like Texas and Florida, have failed to expand Medicaid, experiencing continued declining rural access.^19^ More recently, the One Big Beautiful Bil Act (OBBBA) added cumbersome new work requirements to Medicaid and limited the ways states can finance their programs. As a result, this policy will reduce the number of people covered by Medicaid and increase the level of uncompensated care provided by rural providers, all while decreasing federal Medicaid support for rural providers by $140 billion over the next decade.^20^, ^21^ Also as part of the OBBBA, Senate Republicans passed a $50 billion Rural Health Transformation Program (RHTP) allegedly to stabilize rural health care provision, but providing no fix in practice. The funds from the RHTP can only offset a small percentage of the expect Medicaid losses generally, even less so due to provisions that expressly prohibit much of funds from being used to directly offset Medicaid losses. Moreover, the program provides highly unequal funds based on factors unrelated to the size and need of states’ rural populations, while allowing funds to be siphoned away to nonrural populations.^16^, ^22^, ^23^

Workforce challenges, again stemming from the interaction between rural demographics and insufficient policy support, also plague rural health. Rural Americans have long been less likely to attend college and, thus, medical school and nursing programs, reducing the number of health care professionals with rural backgrounds.^1^, ^15^, ^24^, ^25^ Policymakers sought a remedy for this problem in the ACA by increasing loan repayment assistance for physicians who practice in rural communities and by expanding rural residency opportunities.^26^, ^27^ However, such policies provided too little financial assistance to providers to support residency programs and many individuals who received loan repayments have simply moved away from rural areas once they were unburdened by loans.^15^, ^28^ These workforce shortages are likely to worsen due to the OBBBA, as the Department of Education has moved to remove nursing, physician assistant, public health, and social work degrees from those counted as “professional,” likely reducing the number of people pursuing these careers in areas with already large shortages.^29^, ^30^, ^31^ Enhancements to the Rural Residency Planning and Development grant program or new state programming to provide a “rural administrative subsidy” for participating programs to better cover the costs associated with administering a rural residency program are likely needed to improve such efforts.^28^, ^32^

These and other policy failures have limited the ability of health care providers to respond to rising rates of chronic disease as well as drug addictions and suicide in rural areas.^6^, ^7^, ^8^, ^33^, ^34^ Chronic diseases, like heart disease and Type 2 diabetes, have increased disproportionately in rural areas due to the mixture of limited health access and inadequate policy supports for nutritional assistance, as well as polluted air and unclean water from ineffective environmental regulation.^35^, ^36^, ^37^, ^38^ Similarly, limited mental health care and substance use disorder treatment access in rural areas paired with policy failures to reign in the prescribing practices of physicians or the ease of firearm availability in rural areas have led many rural communities, especially those in economic distress, to be swept away by drug addiction and suicide.^7^, ^8^, ^39^, ^40^, ^41^

These policies failures and the politicians who have strategically politicized them have created a separate policy problem that is also necessary to overcome in the future: the hurdle of an untrusting rural public. Worsening outcomes in rural communities and widening differences between rural and urban places have contributed to rural antipathy toward the government, racialized policy logics, and the withering trust of experts, including the medical profession and specific health policies.^40^, ^42^, ^43^, ^44^, ^45^ Today, largely due to elite policy frames that have sowed doubt and derision, many rural people are opposed to policies and programs that would likely or do help them.^40^ A rural health equity agenda must proceed with these constraints in mind.

## A Policy Agenda for Health Equity in Rural America

There are ample reasons to be pessimistic about the future of rural health. Most health indicators show signs of worsening and many recent policies enacted by the federal government have not helped. However, some federal and state policies, as well as the innovations by health care organizations, offer examples of hope. These interventions have demonstrated an ability to address rural access disparities by providing specialized funding for rural providers, bringing rural people to the care they need, or delivering health care to directly rural people, often utilizing trusted community partners to do so.

Transportation hurdles are often the largest barrier to care.^46^ Most rural areas lack public transportation, which reduces health care utilization.^47^, ^48^ State policies and programs that either subsidize or provide transportation can ease access hurdles. Some states, like Minnesota, Missouri, and New Hampshire, have launched rural transportation cooperatives to do just that.^49^ Policies that provide grants for counties with primary care shortages, that lack hospitals, or to rural‐serving nonprofit hospitals to incentivize coordinated transportation services can help overcome this barrier.

Additionally, telehealth usage in rural areas has expanded considerably, especially since the start of COVID‐19, for services ranging from mental health to substance use disorder treatment to primary care.^50^, ^51^, ^52^ During the pandemic, the federal government modified Medicare via the “Acute Hospital Care at Home” program to allow for more telehealth services to be covered.^53^ Dozens of states have followed suit, expanding the range of telehealth services reimbursed by their Medicaid programs.^54^ However, significant access barriers to broadband itself contributes to lower rural telehealth usage.^55^, ^56^ State policies that expand access to broadband, as well as those that allow for more kinds of health services to be provided via telehealth and reimbursable by Medicaid, can help bridge the accessibility gap.

Promoting the purchasing of mobile health units also offers opportunities to overcome access hurdles. Mobile health units are large recreational vehicle‐like units that can bring health services directly to rural communities.^57^, ^58^ In 2023, the federal government eased the ability of providers to launch new mobile health units under the New Access Point Health Resources and Services Administration grant program via the Maximizing Outcomes through Better Investments in Lifesaving Equipment (MOBILE) Health Care Act.^59^ However, many states continue to have restrictive policies surrounding the permissibility of Medicaid reimbursement for certain services rendered outside of traditional health care facilities.^55^ Expanded grant funding for furnishing and maintaining mobile health units paired with policies that allow for more health services to be reimbursed via these means could further address barriers to care.

Due to the ACA, nonprofit hospitals are required to conduct Community Health Needs Assessments to gauge the broader needs of the communities they serve (e.g., housing, education, food security).^60^ Nonprofit hospitals, which constitute the majority of rural hospitals, have been incentivized under this policy to partner with and support local food banks, religious organizations, educational institutions, and housing authorities to better meet the health needs of the communities they serve.^61^, ^62^ State policymakers should be similarly creative with the potential partnerships available to them to promote rural health. For instance, a federal grant from the Institute of Museum and Library Services enabled Oklahoma to use library resources to help some rural residents access telehealth.^63^ States governments could provide grants to small town libraries or faith‐based organizations to project Wi‐Fi signals further outside buildings, establish and maintain reservable rooms for telehealth, or provide telehealth educational programs to promote telehealth access. Initiatives that leverage existing trusted community members, such as these, may also help overcome the lack of trust many rural people feel toward the government and health care providers.^64^, ^65^, ^66^

These policy initiatives, though helpful, have been modest in scope. Truly maintaining robust health services within rural communities likely requires charting a more heavily government‐backed policy path. Rural health care is ill‐suited for an increasingly profit‐driven health care system, and we must design policies accordingly if we are to keep providers in rural places. The more our health care system has privatized, the more rural communities have been left behind.^67^, ^68^ Strong governmental interventions are what brought modern health care to rural communities in the 20th century, and stronger governmental supports are what is needed to shore up access to care in the 21st.

Short of a complete overall of rural health care financing (e.g., a single‐payer system or a public option), policymakers must reimagine and simplify reimbursement and payment models for public insurers to better subsidize rural care delivery. Private insures pay providers more than what programs like Medicare and Medicaid do. Moreover, rural providers are often paid less by public insurers for the same services delivered at urban facilities, all while managing higher fixed operating costs.^16^ While a variety of special hospital tax designations have been created by policymakers to ease these challenges, they in actuality create complex administrative tasks, underpay rural providers relative to their costs, do not enable rural providers to manage their more sporadic patient volumes, incentivize shedding service lines, and leave out many rural providers due to arbitrary considerations (e.g., being 30 miles from the next closest hospital and not 35).^16^

State and federal governments should adopt a single rural provider designation solely based on the geographic location of providers (e.g., only nonmetropolitan counties) alongside a more robust rural reimbursement rate for Medicare and Medicaid for all providers who meet the new rural designation. One geographically defined designation will reduce administrative burdens for providers, can be used to standardize reimbursements, remove restrictions in care that are required to become qualified for certain designations, and end the practice of urban hospitals gaining rural status to secure special funding under existing policies.^16^, ^69^ A higher rural reimbursement rate would explicitly dispense reimbursements to rural providers that are closer to private insurance to better subsidize the unique costs structures of rural providers, as well as the larger Medicare and Medicaid patient pools and often sicker populations they serve.^70^

Alternative payment models to fee‐for‐service at the federal level from Medicare and state Medicaid programs could also help stabilize rural provider finances. For instance, Mullens, Probst, and Ibrahim^16^ propose the adoption of a “population capitation model,” which would deliver guaranteed funding to hospitals based on the number of potential patients per month in the provider's area. Rural hospital advocates support the adoption of such models, which could make it easier for rural providers to navigate the sporadic amount of care required by their communities.^71^ Alternative payment models in this spirit have been adopted in Maryland to help rural hospital finances.^72^

However, the feasibility of any of these proposals is constrained by contemporary rural politics. Rural communities are often represented by more conservative, Republican politicians who often do not see as important of a role for government in health care.^40^ At the same time, rural people have become less supportive of government initiatives to improve their health and are less trusting of health care professionals, undermining public pressure on politicians to improve rural health access.^40^, ^43^

There is no clearer example of this dynamic than in the politics of Medicaid expansion. Medicaid expansion has had disproportionate rural benefits.^19^, ^73^, ^74^ Indeed, the number of rural hospital closures in states that expanded Medicaid have nearly ended, while they have continued to climb in those that did not.^19^ Despite clear policy benefits, rural people in states that have yet to expanded Medicaid, including those who lost their own hospitals, continue to disapprove of the ACA and Medicaid expansion.^40^, ^45^ To overcome these dynamics, policymakers either need to utilize (and win over) more trusted rural community organizations and institutions to deliver needed improvements, highlight explicitly the “rural only” nature of benefits of policy proposals, and/or secure Republican elite support.^40^ The Republican Party enjoys supermajority support and trust from rural voters and the policies that the Republican Party supports are not met with the same level of public resistance as those from Democrats. Republican buy‐in on rural health policymaking and building rural trust are likely critical for turning the tide on rural health disparities.

There are signs that some in the Republican Party recognize the importance of addressing emergent rural health needs. For instance, in debates over the OBBBA, Republican Senator Josh Hawley called the looming Medicaid cuts as “an attack on our own voters.”^75^ Additionally, in North Carolina, Republican state legislators advocated for Medicaid expansion by stating that the policy will “help our rural hospitals.”^76^ As the rural vote continues to shift toward the Republican Party and as rural health outcomes worsen, Republican politicians may feel increasing pressure to respond to rural health needs. However, given the policies implemented under the second Trump administration to date, whether such a change is coming remains an open question.

## Conclusion

Rural Americans face significant health care access challenges. Communities that are shrinking, aging, and requiring more health care, all while becoming more dependent on public insurers, do not organically make for profitable destinations for health care providers. To meet the needs of rural America today, we must better subsidize health care for rural people. Transportation grants, telehealth, mobile health units, and innovative workforce development policies have demonstrated some success in overcoming rural access struggles. Further investments in these areas can help address the increasing travel burdens and limited local health care accessibility to bring care to rural people or rural people to care.

However, to ensure that health care providers remain and thrive in rural areas, federal and state governments must drastically increase their financial support of rural health providers and further subsidize rural care. Establishing a simple, universal rural provider designation based on geography can ease administrative costs faced and ensure that resources make it to rural communities. Overhauling reimbursement rates to overpay rural providers relative to urban can help these entities navigate the unique operating costs and the less profitable populations they serve. Large government investments were what was required to bring care to rural communities decades ago, and they are what is needed now to keep care within reach of many rural Americans.

## Funding

None.

## Conflict of Interest Statement

None.
